# Impact of hysterosalpingography after operative treatment for ectopic pregnancy in Taiwan: A 16-year Nationwide Population-Based Analysis

**DOI:** 10.1097/MD.0000000000007263

**Published:** 2017-06-23

**Authors:** Nai-Chi Chiu, Chi-Hong Ho, Shu-Huei Shen, Yu-Chuan Tsuei, Kang-Lung Lee, Chen-Yu Huang, Hsin-Yang Li, Tzeng-Ji Chen

**Affiliations:** a Department of Radiology; b Department of Obstetrics and Gynecology, Taipei Veterans General Hospital, Beitou District, Taipei City, Taiwan, R.O.C; and School of Medicine, National Yang-Ming University, Taipei, Taiwan, R.O.C; c School of Medicine, National Yang-Ming University; School of Biomedical Science and Engineering, National Yang-Ming University, Taipei, Taiwan, R.O.C; and Department of Orthopaedics, Cheng Hsin General Hospital, Taipei City, Taiwan R.O.C; d Department of Family Medicine, Taipei Veterans General Hospital, Taipei City, Taiwan, R.O.C; and Institute of Hospital and Health Care Administration, School of Medicine, National Yang-Ming University, Taipei, Taiwan, R.O.C.

**Keywords:** ectopic pregnancy, hysterosalpingography, Taiwan

## Abstract

By retrieving records from Taiwan's National Health Insurance (NHI) system's database, the current study aimed to investigate the impacts of hysterosalpingography (HSG) to patients after ectopic pregnancy (EP) operations in Taiwan.

In this retrospective cohort study, insurance claims data from 1997 to 2013, derived from a cohort of 1 million people randomly sampled to represent all NHI beneficiaries, were analyzed. Patients after ectopic pregnancy (EP) operations were identified via the inclusion of the corresponding NHI procedure codes. We further divided the patients into 2 groups by whether received subsequent HSG, EP-HSG, and EP-no-HSG. Patients with history of previous pregnancies (PP) and subsequent HSG were grouped as PP-HSG. We sought to evaluate the following pregnancies (FP) rate, interval to FP in EP-HSG compared with that in EP-no-HSG, and PP-HSG.

EP-HSG had significantly higher FP rate odds ratio than EP-no-HSG (OR, 1.64; 95% CI, 1.24–2.16, *P* < .001). EP-HSG had lower FP rate odds ratio than that in PP-HSG, but no significant difference (33.1% vs 34.6%, *P*  =  .654). The INTERVAL_(HSG-FP)_ in EP-HSG was no significantly different from that in PP-HSG (843.34 ± 82 days vs 644.72 ± 24.30 days, *P*  =  .077). There was significant positive correlation between FP after EP and number of HSG (*r*  =  0.070^∗∗^, *P* < .001). There were significant negative correlation between FP and EP age (*r*  =  −0.270^∗∗^, *P* < .001), FP and INTERVAL_(EP-HSG)_ (*r*  =  −0.212^∗∗^, *P*  =  .001). The multivariate analysis showed that INTERVAL_(EP-HSG)_ less than 1 year is the predictor factor of INTERVAL_(EP-FP)_ (hazard ratio: 1.422; 95% CI: 1.130–1.788; *P* *=* .003). It was evident that the longer the INTERVAL_(EP-HSG)_, the lower the FP rate odds ratio; and the older the EP age, the lower the FP rate odds ratio. (OR, 95% CI; >1 year: 0.59, 0.41–0.86; >2 year: 0.42, 0.32–0.55; >25 years old: 0.47, 0.38–0.57; >30 years old: 0.29, 0.24–0.35; >35 years old: 0.12, 0.08–0.18, all *P* < .001).

Receiving HSG after EP, short INTERVAL_(EP-HSG)_, EP age less than 30 years old, had significant positive impacts on the FP. We encourage shortening the INTERVAL_(EP-HSG)_, and the counseling of women on the most appropriate way to conceive thereafter.

## 1 Introduction

Taiwan's birthrate is one of the lowest in the world, and the total female fertility rate fell from 1.68 in 2000 to 1.18 in 2015,^[1]^ a decrease of 43%. In Taiwan, an increasing number of couples are seeking medical treatment for infertility.^[2]^ Female fertility decreases gradually with age, significantly declining from approximately the age of 32 years and decreasing more rapidly after the age of 37.^[3]^ Considering the anticipated age-related decline in fertility, the increased incidence of disorders that impair fertility, and the higher risk of pregnancy loss, women older than 35 years who have failed to conceive for 6 months should receive an expedited evaluation, and undergo treatment if clinically indicated.^[3]^

Ectopic pregnancy (EP) is defined as the abnormal implantation of an embryo outside the uterine endometrium. The most common site of EP is a fallopian tube.^[4]^ There have been an increasing number of EP cases detected in recent years, due to improved earlier diagnostic techniques. The risk factors for EP include pelvic inflammatory disease, cigarette smoking, assisted reproductive techniques (ARTs), and caesarian sections.^[5,6]^ Endometriosis is the largest risk factor for EP in Taiwan.^[7,8]^

The treatments for EP include methotrexate, conservative surgery (salpingostomy, salpingotomy), and radical surgery (salpingectomy). The consensus on the management of severe EPs is that they require a surgical approach.^[9]^ Early diagnosis of EP and better access to care have shifted concern to the issue of preserving subsequent fertility. Fertility is compromised in women whose first pregnancy is ectopic. Well-developed ARTs could improve long-term delivery rates in women with EPs.^[10]^

Hysterosalpingography (HSG) plays a crucial role in infertility evaluation. It determines the anatomic causes of female subfertility and/or infertility, especially for uterine structure and tubal status abnormalities. HSG has high reproducibility^[11]^ and is of significant value for evaluating tubal patency after treatment for an EP.^[12]^

The primary aim of this study was to retrospectively evaluate the impacts of subsequent HSG after surgical treatment for an EP (EP-HSG), and compare the following pregnancies (FP) with those had no subsequent HSG after EP (EP-no-HSG). The secondary aim of the study was to compare the FP between patients had HSG after previous pregnancies (PP-HSG) and those had HSG after EP (EP-HSG).

## 2 Methods

### 2.1 Database

The NHI program is the sole provider of health insurance in Taiwan. It was launched in 1996, and as of 2016, more than 99.6% of the Taiwanese population were enrolled in it.^[13]^ The NHI research database (NHIRD), which contains NHI claims data, is updated by the National Health Research Institutes each year. Personal identification information is encrypted before the release of the research database to protect patient privacy. The study was conducted in accordance with the Declaration of Helsinki and was approved by the institutional review board of Taipei Veterans General Hospital according to Republic of China law (VGHIRB No.: 2013–04–005E). A cohort dataset of 1 million people randomly sampled to represent all NHI beneficiaries was used. (Longitudinal Health Insurance Database 2000, LHID2000). LHID2000 was randomly selected from the 23 million beneficiaries of the National Health Insurance Research Database (NHIRD) in Taiwan. Both hospitalization and ambulatory records, including the encrypted personal identification number, date of birth, gender, procedure code as defined in the fee schedule and reference list for medical services of the NHI, and the specialty of the physician in charge were analyzed.

### 2.2 Study population

**Figure 1:**
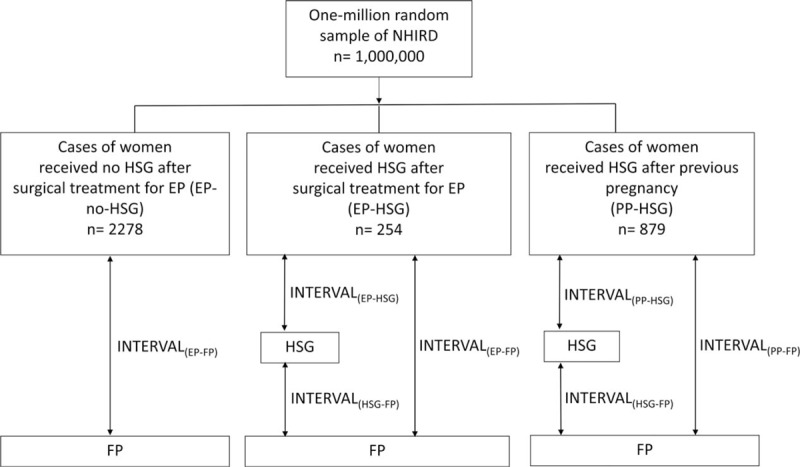
Flowchart for the recruitment of subjects from the 1 million random individuals in the National Health Insurance Research Data-base (NHIRD) from 1997 to 2013 in Taiwan. NHIRD  =  National Health Insurance Research Database.

Insurance claims data from 1997 to 2013 were used in this study. Figure 1 shows the flowchart for the selection of study population. Of the initial 1 million individuals, we excluded 34 subjects because of missing data regarding age and sex. The diagnoses used to identify patients with EPs included the following codes from The International Classification of Diseases, Ninth Revision, Clinical Modification: 633 (ectopic pregnancy), 633.0 (abdominal pregnancy), 633.1 (tubal pregnancy), 633.2 (ovarian pregnancy), 633.8 (other ectopic pregnancy), and 633.9 (unspecified ectopic pregnancy). Surgical approaches to EP included salpingotomy (code 66.01), salpingostomy (code 66.02), salpingectomy with removal of a tubal pregnancy (code 66.62), and removal of an EP (code 74.3). Patients who received HSG were identified via the inclusion of the NHI procedure code 33029B in their medical records. The diagnoses used to identify patients with PP and FP included the following codes: 97001K (normal spontaneous delivery) and 97006K (cesarean section). Patients with a diagnosis of EP who received surgical procedures were included. We further divided the EP patients into 2 groups by whether received HSG within the subsequent 10 years, EP-HSG group and EP-no-HSG group. Patients with history of previous pregnancies (PP) and subsequent HSG were identified as the PP-HSG group. Utilization rates were calculated per 1000 beneficiaries.

### 2.3 Statistical analysis

The continuous values in our study were measured by coefficient of skewness (β1) and coefficient of kurtosis (β2). The clinical characteristics of both groups were compared by using a Mann–Whitney *U* test for continuous values and Pearson's chi-squared analysis or Fisher's exact test for proportions. Continuous variables were presented as means and standard errors. The interval from EP to HSG (INTERVAL_(EP-HSG)_) was calculated from the date of receiving operations for EP to the date of receiving HSG. The interval from PP to HSG (INTERVAL_(PP-HSG)_) was calculated from the date of previous normal spontaneous delivery or receiving cesarean section to the date of receiving HSG. The interval from HSG to FP (INTERVAL_(HSG-FP_) was calculated from the date of receiving HSG to the date of subsequent normal spontaneous delivery or receiving cesarean section. The interval from EP to FP (INTERVAL_(EP-FP)_) was calculated from the date of receiving operations for EP to the date of subsequent normal spontaneous delivery or receiving cesarean section. The interval from PP to FP (INTERVAL_(PP-FP)_) was calculated from the date of previous normal spontaneous delivery or receiving cesarean section to the date of subsequent normal spontaneous delivery or receiving cesarean section. The INTERVAL_(EP-FP)_ and INTERVAL_(PP-FP)_ was estimated by the Kaplan–Meier method. A univariate Cox regression analysis of the clustered data was used to test for the association between the baseline characteristics and the INTERVAL_(EP-FP)_ and INTERVAL_(PP-FP)_. A multivariate analysis conducted by using a Cox proportional hazards model for the clustered data was performed to identify predictive variables while adjusting for the other characteristics. All variables were included in the full model, and the parameter estimates for this full model are provided. Hazard ratios (HRs) and the corresponding 95% confidence intervals (CIs) were reported. Variables with statistical significance (*P* < .05) or proximate to it (*P* < .1) in the univariate analysis were included in the multivariate analysis via a forward stepwise Cox regression model. Effects were calculated in terms of odds ratios (ORs) and the corresponding 95% confidence intervals. Correlation between FP and clinical parameters was obtained using Spearman's correlation coefficient for statistical analysis. A 2-tailed *P* < .05 was considered statistically significant. All statistical analyses were performed using IBM SPSS Statistics for Windows, version 21.0 (IBM Corp., Armonk, NY). Data management and collection were conducted using PostgreSQL version 9.34 (PostgreSQL Global Development Group).

## 3 Results

**Figure 2:**
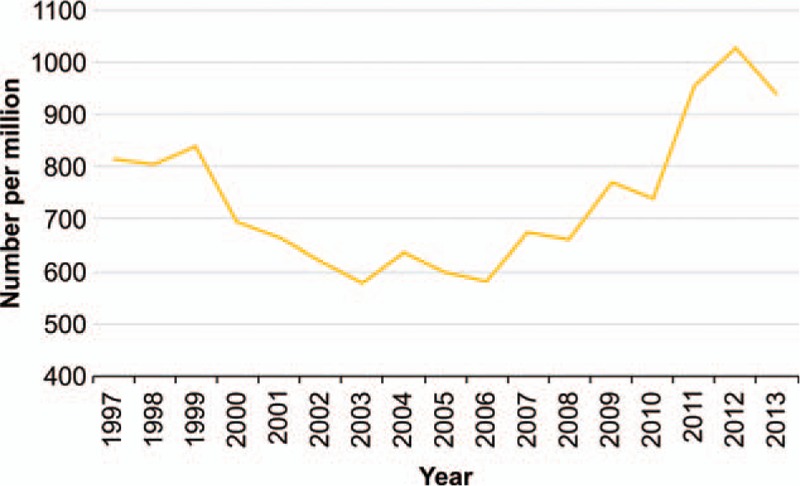
Total numbers of HSG procedures recorded from 1997 to 2013 in a cohort of 1 million people randomly sampled from Taiwan's National Health Insurance Research database. HSG  =  hysterosalpingography.

Based on the sampling data, the total number of HSGs increased 178% from 2003 to 2012 (Fig. 2). There was a sharp acceleration in this increase from 2010 to 2011. A total of 2532 women having surgical treatment for EP were identified. There were 2278 cases of women received no HSG after surgical treatment for EP (EP-no-HSG), 254 cases of women received HSG after surgical treatment for EP (EP-HSG), 879 cases of women received HSG after previous pregnancy (PP-HSG).

### 3.1 Comparison of clinical demographics for EP-no-HSG and EP-HSG

FP rate in the EP-HSG group was significantly higher than that in the EP-no-HSG group (33.1% vs 23.2%, *P*  =  .002) (Table 1). Number of FP in the EP-HSG group was significantly higher than that in the EP-no-HSG group (0.45 ± 0.04 versus 0.30 ± 0.13, *P* < .001).

**Table 1:**
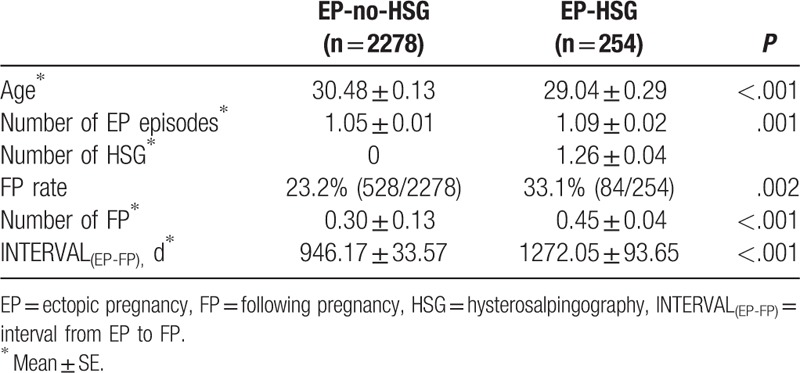
Comparison of the demographic data of EP-no-HSG (n  =  2278) and EP-HSG (n  =  254).

### 3.2 Comparison of clinical demographics for PP-HSG and EP-HSG

Age of PP was significantly younger than age of EP (27.33 ± 0.18 vs 29.04 ± 0.29, *P* < .001) (Table 2). The number of received HSG in the EP-HSG group was significantly higher than that in the PP-HSG group (1.26 ± 0.04 versus 1.18 ± 0.03, *P*  =  .004). INTERVAL_(PP-HSG)_ was significantly longer than INTERVAL_(EP-HSG)_ (1728.80 ± 38.00 days vs 718.14 ± 43.04 days, *P* < .001). INTERVAL_(HSG-FP)_ was no significantly different in 2 groups (PP-HSG: 644.72 ± 24.30 days; EP-HSG: 843.34 ± 82.71, *P*  =  .077). The FP rate was no significantly different in 2 groups (PP-HSG: 34.6%; EP-HSG: 33.1%, *P*  =  .654). The number of FP was no significantly different in 2 groups (PP-HSG: 0.39 ± 0.02; EP-HSG: 0.45 ± 0.04, *P*  =  .857).

**Table 2:**
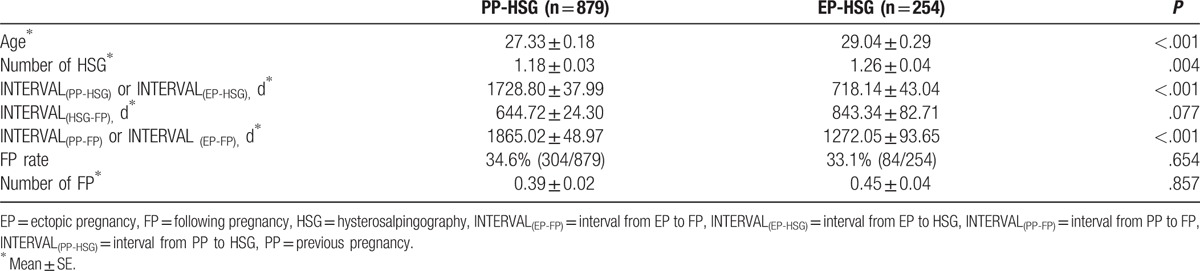
Comparison of the demographic data of PP-HSG (n  =  879) and EP-HSG (n  =  254).

### 3.3 Correlation between FP and clinical parameters

There was significant positive correlation between FP and number of HSG (*r*  =  0.070^∗∗^, *P* < .001) (Table 3). There were significant negative correlation between FP and EP age (*r*  =  −0.270^∗∗^, *P* < .001), between FP and INTERVAL_(EP-HSG)_ (*r*  =  −0.212^∗∗^, *P*  =  .001), between FP and PP age (*r*  =  −0.072^∗^, *P*  =  .034), between FP and INTERVAL_(PP-HSG)_ (*r*  =  −0.355^∗∗^, *P* < .001).

**Table 3:**
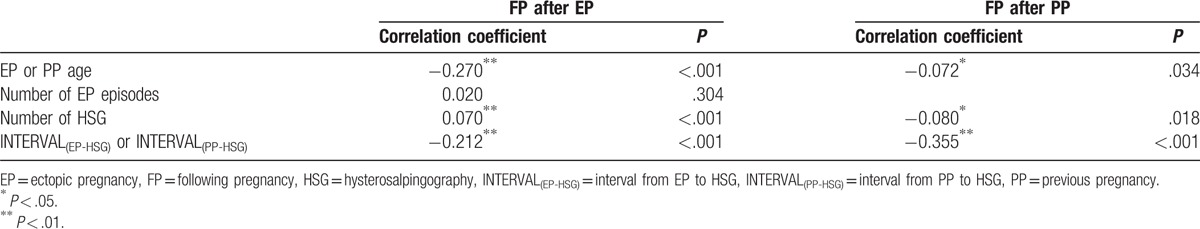
Correlation between FP and clinical variables, obtained using Spearman's correlation coefficient for statistical analysis.

### 3.4 Correlation between INTERVAL_(EP-FP)_ and clinical parameters

There were significant positive correlations between number of HSG and INTERVAL_(EP-FP)_ (*r*  =  0.176, *P* < .001), between INTERVAL_(EP-HSG)_ and INTERVAL_(EP-FP)_ (*r*  =  0.411, *P* < .001), between number of HSG and INTERVAL_(PP-FP)_ (*r*  =  0.123, *P*  =  .032), between INTERVAL_(PP-HSG)_ and INTERVAL_(PP-FP)_ (*r*  =  0.834, *P* < .001) (Table 4). There were significant negative correlations between age of EP and INTERVAL_(EP-FP)_ (*r*  =  −0.206, *P* < .001), and between age of PP and INTERVAL_(PP-FP)_ (*r*  =  −0.251, *P* < .001).

**Table 4:**
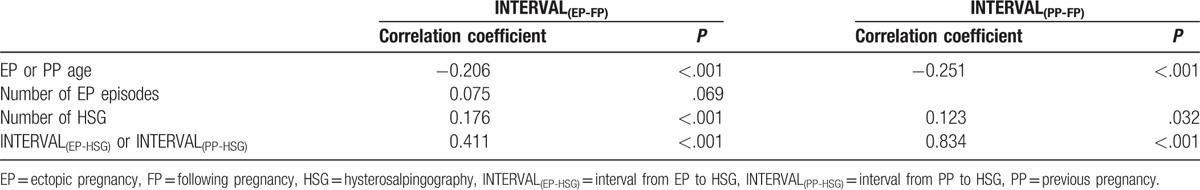
Correlation between INTERVAL_(EP-HSG)_ or INTERVAL_(PP-HSG)_ and clinical variables, obtained using Spearman's correlation coefficient for statistical analysis.

### 3.5 Factors associated with INTERVAL_(EP-FP)_ and INTERVAL_(PP-FP)_

In the univariate analysis, factors predict the INTERVAL_(EP-FP)_ included EP age less than 30 years old (*P* < .001), number of HSG (*P*  =  .005), and INTERVAL_(EP-HSG)_ less than 1 year (*P*  =  .006) (Tables 5 and 6). In the stepwise multivariate analysis, EP age less than 30 years old and INTERVAL_(EP-HSG)_ less than 1 year were the significant predictors of INTERVAL_(EP-FP)_. The multivariate analysis showed that INTERVAL_(PP-HSG)_ less than 1 year was the significant predictor of INTERVAL_(PP-FP)_. In the age matched multivariate analysis, the interval of HSG after PP or EP was the significant predictor of FP (HR, 1.975; 95% CI, 1.559–2.501, *P* < .001).

**Table 5:**
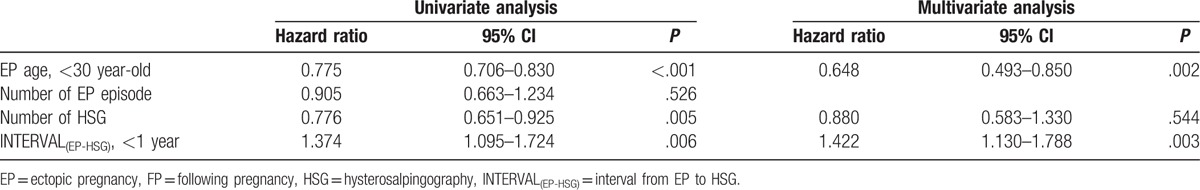
Multivariate analysis of predict factors for INTERVAL_(EP-FP)._

**Table 6:**
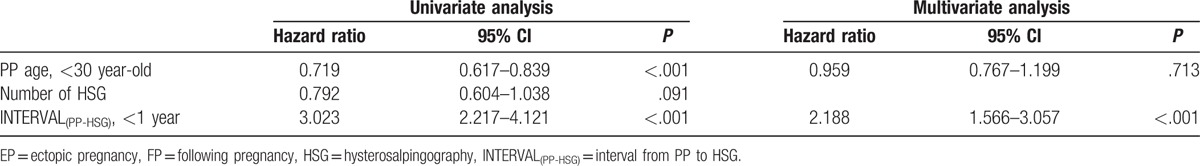
Multivariate analysis of predict factors for INTERVAL_(PP-FP)._

### 3.6 HSG impacts on FP rate estimated in terms of odds ratios

EP-HSG had significantly higher FP odds ration than EP-no-HSG (odds ration OR, 1.64; 95% CI, 1.24–2.16, *P* < .001) (Table 7). EP-HSG had lower FP odds ratio than PP-HSG, but no significant difference (OR, 0.76; 95% CI, 0.56–1.04, *P*  =  .08). Our study showed that the longer the INTERVAL_(EP-HSG)_, the lower the FP odds ratio (more than 1 year: OR, 0.59; 95% CI, 0.41–0.86, *P* < .001; more than 2 year: OR, 0.42; 95% CI, 0.32–0.55, *P* < .001). Another evidence was that the older the EP age the lower the FP odds ratio (>25 years old: OR, 0.47; 95% CI, 0.38–0.57, *P* < .001; >30 years old: OR, 0.29; 95% CI, 0.24–0.35, *P* < .001; >35 years old: OR, 0.12; 95% CI, 0.08–0.18, *P* < .001).

**Table 7:**
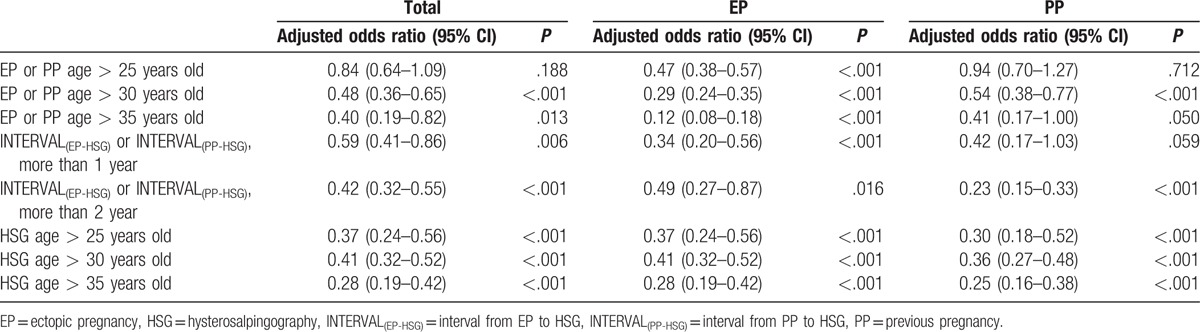
HSG impacts on the FP rate estimated in terms of odds ratios.

## 4 Discussion

The present study is an important large-scale survey of receiving HSG after operative treatment for EP in a Taiwanese population. Based on the sampling data, HSG after EP had significant impacts to the FP.

In 1985, Taiwan's first in vitro fertilization was born. The event remained in the news headlines in all of the major newspapers for an entire week. ART was a reflection of Taiwan's national power in achieving medical miracles in the early 1980s. The pressure for Taiwan to achieve an “in vitro fertilization” increased after successful cases in Singapore and Japan.^[17]^ Based on the sampling data, the total number of HSGs has increased gradually since 2006. There was a sharp acceleration of the total number of HSGs from 2010 to 2011. While we do not know the precise reason for this observation, traditionally, most Chinese individuals like to have babies in a “Year of the Dragon” (a “Dragon Year Baby”), and 2012 was such a year. Although there was a small drop in the number of HSGs from 2012 to 2013, we believe that the total number of HSGs will continue to increase in the future.

In Taiwan, there have been considerable changes in the surgical approaches to EP treatment in recent decades, specifically a shift from laparotomy to laparoscopy.^[14]^ The most recent and thorough results from the DEMETER randomized trial suggest that there was no significant difference in subsequent 2-year following pregnancy (FP) when comparing conservative surgery (salpingotomy) to salpingectomy (70% vs 64%, hazard ratio, 1.06; CI, .69–1.63; *P*  =  .78).^[15]^ HSG is the standard first-line test to evaluate tubal pregnancy. It also has a therapeutic effect.^[16]^ HSG is of significant value for evaluating tubal patency after treatment for an EP. HSG results following EP treatment are significantly associated with subsequent spontaneous pregnancy rates.^[12]^ Based on our study, the FP rate was no significant difference between PP-HSG and EP-HSG, but significant difference between EP-no-HSG and EP-HSG. HSG had significant impacts on the FP in both PP-HSG and EP-HSG.

Female fertility decreases gradually with age, significantly declining from approximately the age of 32 years.^[3]^ Based on our study, although there was a trend that, the older the EP age and the longer the INTERVAL_(EP-HSG)_, the lower the FP odds ratio. However, HSG had promising impacts on FP. The INTERVAL_(HSG-FP)_ was no significantly different in EP-HSG and PP-HSG. Thus, we suggest shortening the INTERVAL_(EP-HSG)_ to those who have planning for future pregnancy.

Neither methotrexate, nor conservative surgery (salpingotomy) or radical surgery (salpingectomy) for EP treatment significantly affect ovarian function.^[17,18]^ Long-term following pregnancy (FP) among women with a first EP have improved over time. According to our nationwide population base study, the FP rate in EP-HSG was 33.1%, significantly higher than that in EP-no-HSG (23.2%, *P*  =  .002).

There are limitations to the present study. First, we recruited patients who received surgeries for EP, which might have led to selection bias and limited external validity of the findings. Second, this was a retrospective observational study and a randomized clinical trial is required to validate our findings. The total number of patients received HSG after received operative treatment for EP was relative small. However, the NHIRD is a very complete database, which includes a large sample size of subjects. The analyses results of the NHIRD are reliable and provide valid information regarding patients’ medical-seeking behavior in Taiwan.

In conclusion, receiving HSG after EP, short INTERVAL_(EP-HSG)_, EP age less than 30 years old, had significant positive impacts on the FP. Female fertility decreases gradually with age. Considering the age-related decline in fertility, the increased incidence of disorders that impair fertility, and the higher risk of pregnancy loss, we suggest an expedited evaluation for those who received operative treatment for EP. Education and enhanced awareness of the effect of age on fertility are essential in counseling women desiring to become pregnant. Taiwan has well-developed ARTs and facilities. We encourage shortening the INTERVAL_(EP-HSG)_ and the counseling of women on the most appropriate way to conceive thereafter.

## Acknowledgments

Authors would like to thank Yu-Chi Cheng, Fu-Chieh Hsu, and Hsieh-Chih Chen, who assisted us with the study design and data mining. They would also like to thank Editage (www.editage.com) for English language editing and publication support. No funding was received for this research. All authors declare that there are no conflicts of interest. They did not have unlabeled use of products in this study.
